# Examining the association between neuropsychiatric symptoms among people with dementia and caregiver mental health: are caregiver burden and affiliate stigma mediators?

**DOI:** 10.1186/s12877-023-03735-2

**Published:** 2023-01-16

**Authors:** Yi-Jung Chen, Jian-An Su, Jung-Sheng Chen, Chieh-hsiu Liu, Mark D. Griffiths, Hsin-Chi Tsai, Chih-Cheng Chang, Chung-Ying Lin

**Affiliations:** 1 Institute of Allied Health Sciences, College of Medicine, National Cheng Kung University, Tainan, Taiwan; 2 Department of Psychiatry, Chang Gung Medical Foundation, Chiayi Chang Gung Memorial Hospital, Chiayi, Taiwan; 3 School of Medicine, Chang Gung University, Taoyuan, Taiwan; 4 Department of Nursing, Chang Gung Institute of Technology, Taoyuan, Taiwan; 5 Department of Medical Research, E-Da Hospital, I-Shou University, Kaohsiung, 82445 Taiwan; 6 Department of Family Medicine, Taoyuan General Hospital, Ministry of Health and Welfare, Taoyuan, Taiwan; 7 International Gaming Research Unit, Psychology Department, Nottingham Trent University, Nottingham, UK; 8 Department of Psychiatry, School of Medicine, Tzu Chi University, Hualien, Taiwan; 9 Department of Psychiatry, Tzu-Chi General Hospital, Hualien, Taiwan; 10 Department of Psychiatry, Chi Mei Medical Center, Tainan, Taiwan; 11 Department of Health Psychology, Chang Jung Christian University, Tainan, Taiwan; 12 Biostatistics Consulting Center, National Cheng Kung University Hospital, College of Medicine, National Cheng Kung University, Tainan, Taiwan; 13 Department of Occupational Therapy, College of Medicine, National Cheng Kung University, Tainan, Taiwan; 14 Department of Public Health, National Cheng Kung University Hospital, College of Medicine, National Cheng Kung University, Tainan, Taiwan

**Keywords:** Affiliate stigma, Burden, Caregiver, Dementia, Mediation

## Abstract

**Background:**

Neuropsychiatric disturbances are common manifestations of dementia disorders and are associated with caregiver burden and affiliate stigma. The present study investigated affiliate stigma and caregiver burden as mediators for the association between neuropsychiatric symptoms of people with dementia (PWD) and caregiver mental health such as depression and anxiety.

**Methods:**

A cross-sectional survey study was carried out with 261 dyads of PWD and informal caregivers from the outpatient department of a general hospital in Taiwan. The survey included the Caregiver Burden Inventory (CBI), the Affiliate Stigma Scale (ASS), the Taiwanese Depression Questionnaire (TPQ), and the Beck Anxiety Inventory (BAI). Mediation models were tested using the Hayes’ PROCESS macro (Model 4 for parallel mediation model; Model 6 for sequentially mediation model).

**Results:**

Caregiver burden, affiliate stigma, caregiver depression, and caregiver anxiety were significantly associated with neuropsychiatric symptoms. After controlling for several potentially confounding variables, it was found that PWD’s neuropsychiatric symptoms, caregiver burden and affiliate stigma significantly explained 52.34% of the variance in caregiver depression and 37.72% of the variance in caregiver anxiety. The parallel mediation model indicated a significantly indirect path from PWD’s neuropsychiatric symptoms to caregiver mental health through caregiver burden and affiliate stigma, while the direct effect was not significant. Moreover, there was a directional association between caregiver burden and affiliate stigma in the sequential mediation model.

**Conclusions:**

These findings show that it is imperative to improve caregivers’ perception of those with dementia to reduce internalized stigma and to improve caregivers’ mental health. Implementation of affiliate stigma assessment in clinical practice would allow distinctions to be made between the impact of affiliate stigma and the consequences of caregiver burden to help inform appropriate intervention.

## Introduction

According to the World Alzheimer Report [1], approximately 46.8 million individuals are living with dementia globally. The number has not plateaued and it is predicted that it will increase to 131.5 million individuals by the year 2050 [2]. Therefore, large demands on caring for older people, especially those with dementia, are needed and such burden is usually relied on informal caregivers. Informal caregivers have been viewed as invisible second patients [3]. Indeed, providing care to a family member with disease can cause emotional, physical, and financial burden to the informal caregivers [4–6]. Among caregivers taking care of family member with different types of disease, those taking care of people with dementia (PWD) appear to have greater levels of psychological burden. Some research has reported that caregivers of patients with Alzheimer’s disease (AD) have a higher prevalence of depression and anxiety than caring for patients with other illnesses [6–8]. A meta-analysis comprising 17 studies (*N* = 10,825 participants) reported a high prevalence of depression (34.0%) and anxiety (43.6%) for caregivers of patients with AD [9]. In Taiwan (where the present study was carried out), similar prevalence rates have been reported: 23.7–43.8% at risk of depression and 37.4% at risk of anxiety among informal caregivers of PWD [10, 11]. Therefore, the mental health of caregivers who take care of PWD should also be taken care of by healthcare providers.

The model proposed by Pearlin et al. [12] provides a potential psychological framework for healthcare providers to tackle mental health of caregivers of PWD. More specifically, the Stress Process Model (SPM) comprises the caregiving context (e.g., social and economic characteristics). Also, during the process, stressors (including objective indicators such as problematic behavior and subjective indicators such as burnout felt by caregivers) lead to psychological manifestations (e.g., depression, anxiety) via some mediators such as coping strategies [12]. Therefore, behavioral disturbances, particularly angry or aggressive behaviors among PWD, are objective stressors associated with caregiver depression [13–15]. Similarly, caring for PWD increases caregiving burden, a type of subjective stressor, and such subjective burden also increases the risk of having depression and anxiety symptoms [16]. In sum, behavioral and psychological symptoms of dementia (e.g., irritability, agitation, aggression, apathy) together with the caregivers’ caregiving burden are potential stressors that can result in impaired mental health among caregivers of PWD [15, 17].

The burden experienced by caregivers is a result of many factors. As individuals gradually lose the ability to care for themselves, there is increasing need for supervision and assistance. Novak et al. [18] have identified five dimensions of subjective burden comprising (i) time-dependence burden (i.e., time cost of the caregiver), (ii) developmental burden (i.e., the caregivers’ feelings of being ‘off-time’ in their development with respect to their peers [e.g., missing out on what others do because of their caring duties]), (iii) physical burden (i.e., caregivers’ feelings of chronic fatigue and damage to their physical health, (iv) social burden (i.e., caregivers’ feelings of role conflict), and (v) emotional burden (i.e., caregivers’ negative feelings toward their care receivers). The five dimensions of subjective burden may result from the individual’s unpredictable and often bizarre behavior [18, 19]. Studies of caregiver burden have also shown that increases in the care-receiver’s behavioral and psychological symptoms are strongly correlated with caregiver burden [15, 20–22]. Moreover, care-receiver’s behavioral and psychological symptoms (e.g., hallucinations, irritability, depression) are significant predictors of caregiver burden [17].

One study by Werner et al. assessed four dimensions of affiliate stigma—interpersonal interaction, concealment, structural discrimination, and access to social roles conducted [23]. Caregivers of people with Alzheimer’s disease have especially high levels of affiliate stigma in the four aforementioned dimensions. Such affiliate stigma prevents informal caregivers from seeking the services that might reduce caregiver burden. Other family members may blame informal caregivers for providing a poor home environment or mismanaging PWD [24]. Informal caregivers may lose their jobs due to managing symptoms of PWD in emergency situations related to their wandering, falls, and basic needs. These may include employment discrimination or other forms of structural discrimination as well as loss of social relationships and experiences of harsh social judgments [25]. Therefore, caregivers may internalize negative stereotypes from the social stigma, resulting in affiliate stigma. In brief, affiliate stigma is a type of internalized stigma (i.e., the caregivers internalize the stigma themselves because of their relationship with PWD), and the negative effects of internalized stigma on mental health of stigmatized populations have been widely reported [26–28].

From the information mentioned above, affiliate stigma is another important factor that could contribute to caregiving burden and psychological distress of caregivers of PWD. Many caregivers suffer from stigma experiences because of their family member’s mental illness (i.e., courtesy stigma as defined by Goffman [29]), and such stigma experiences may be internalized by the caregivers and become affiliate stigma [26]. In other words, when a society treats the caregivers of PWD with negative perceptions, attitudes, emotions, and avoidant behaviors, caregivers are at risk of having negative experiences in emotional (e.g., anxiety), social (e.g., family burden) and interpersonal (e.g., isolation) aspects [30]. Indeed, empirical evidence has shown strong associations between affiliate stigma among informal caregivers and negative outcomes, including caregiver burden [11, 31], quality of life [32], depression [27, 28], anxiety [27, 28, 31].

In order to provide high quality programs to improve the mental health of caregivers who take care of PWD, it is crucial for healthcare providers to better understand the psychological mechanisms that underpin their psychological distress such as depression and anxiety. More specifically, different factors (e.g., PWD’s clinical characteristics and caregivers’ demographics) should be tested to yield the most important factors for healthcare providers to foster an efficient program. The best way to investigate the psychological mechanisms is to use a well-established theory or model. Therefore, the present study was guided by the SPM and proposes two mediation models (a parallel mediation model and a sequential mediation model). More specifically, the present study proposed and tested the mediating role of affiliate stigma and caregiver burden after controlling for several confounding variables associated with the PWD’s behavioral and psychological symptoms (e.g., PWD’s age, sex, marital status, employment status, education and relationship of caregiver, and patient’s age, sex, marital status). The present study’s simplified SPM retains the following factors derived from the original SPM: background information (treated as the confounding variables); primary stressors, including behavioral and psychological symptoms (treated as the independent variables), affiliate stigma, and caregiver burden (treated as the mediator); and depression and anxiety (treated as the outcome). While the PWD’s conditions cannot be changed, reducing affiliate stigma or caregiver burden could be effective in improving caregiver mental health if either of them was found to be a significant mediator. Furthermore, by separating stressors into affiliate stigma and caregiver burden, the present study also addresses the question of which approach caregiver support services should be more emphasized.

To the best of the present authors’ knowledge, no empirical evidence has been reported regarding whether the SPM could be an effective model in explaining mental health consequences among caregivers of PWD. Therefore, the present study sought to address this knowledge gap in the literature on PWD through two types of mediation model examining the severity of neuropsychiatric symptoms that affect caregiver depression and anxiety. It was hypothesized that caregivers experiencing increasing levels of affiliate stigma would be more likely to report higher levels of depression and anxiety, and that the relationship between neuropsychiatric symptoms and caregiver mental health would be mediated by caregiver burden and affiliate stigma.

## Methods

### Participants and data collection

A cross-sectional survey study utilizing convenience sampling was designed to collect data from a dementia care center at a general hospital located in Southern Taiwan. 300 caregivers who took care of the patients with dementia at home were invited to participate in the study. More specifically, several psychiatrists in the dementia care center screened potential participants (please see the inclusion and exclusion criteria below for details) when the informal caregivers and the PWD visited the dementia care center for daycare service. Then, the psychiatrists transferred the eligible participants to several research assistants to further confirm the eligibility of the participants. When the eligibility was confirmed, the research assistants led the participants to a quiet room to complete the self-reported measures using pen and paper. After excluding those who had incomplete data (*n* = 39), the final data used for analyses included 261 patient-caregiver dyads (i.e., 87% of response rate). All the participants (i.e., both caregivers and patients) provided their informed consent after being told of the study purpose. For those who had severely impaired cognitive capacity, consent was obtained from their legally authorized representatives following assessment by their treating psychiatrist(s). The study’s inclusion criteria were: (i) ability to speak, understand and read Mandarin Chinese or Taiwanese, (ii) each caregiver participant had at least one family member aged older than 65 years (because young-onset dementia is conventionally thought to include patients with onset before the age of 65 years [33], and this cutoff point is indicative of a sociological partition in terms of employment and retirement age) with any type of diagnosed dementia (including AD and vascular dementia), and (iii) each caregiver participant was aged at least 20 years because the Civil Law in Taiwan sets 20 years as the age of adulthood. Caregivers who were diagnosed with a mental illness or had problems in understanding the survey questions were excluded. All procedures used for the present study were approved by the institutional review board of the Chang Gung Memorial Hospital (IRB 102-3378B).

## Measures

In the present study, all assessment items asked the current condition of each participant, except for those assessing depression and anxiety which concerned the condition over the past week. In addition, demographic information concerning the caregivers was collected (i.e., age, sex, years of education, marital status, employment status, relationship to the person with dementia) and the demographic variables for the person with dementia they cared for (i.e., age, sex, marital status, and neuropsychiatric symptoms).

### Caregiver Burden Inventory (CBI)

The 24-item Chinese version of the Caregiver Burden Inventory (CBI) was used to assess caregivers’ burden in taking care of PWD. The CBI also provides a brief and comprehensive measure of caregiver burden that makes it a practical tool for assessing and responding to caregiver burden [19]. The CBI has five burden subscales (time-dependent = 5 items, developmental = 5 items, physical = 4 items, social = 4 items, and emotional = 6 items). Each CBI item is rated on a 5-point Likert scale ranging from 0 (strongly disagree) to 4 (strongly agree). The total CBI score ranges from 0 to 96, where a higher score indicates a higher the level of caregiver burden [18]. The overall scale has been shown to have excellent internal consistency (Cronbach’s α = 0.91) [19].

### Affiliate Stigma Scale (ASS)

The 22-item Chinese version of the Affiliate Stigma Scale (ASS) was used to assess caregivers’ internalization of stigma [26]. This theory driven instrument was designed for easy and practical use in clinical settings due to the fewer items, and its appropriateness for a wide range of family caregivers including children, spouses, grandchildren, and other relatives [27, 28]. The ASS has three subscales (cognitive = 7 items, affect = 7 items, and behavior = 8 items). Each ASS item is rated on a 4-point Likert scale ranging from 1 (strongly disagree) to 4 (strongly agree). The total ASS score ranges from 22 to 88, where a higher score indicates a higher the level of affiliate stigma. The psychometric properties of the ASS have been tested for caregivers of family members with dementia and has been shown to have very good internal consistency (Cronbach’s α = 0.82–0.85) [27].

### Taiwanese Depression Questionnaire (TDQ)

The 18-item Taiwanese Depression Questionnaire (TDQ) was used to assess caregivers’ depressive symptoms [34]. The TDQ was constructed for experiences among the Taiwanese population. Consequently, the TDQ has been widely used as a screening tool for studies conducted in Taiwan [35]. Each TDQ item is rated on a 4-point Likert scale ranging from 0 (no or extremely few, < 1 day per week) to 3 (often or always, 5–7 days per week). The total TDQ score ranges from 0 to 54, where a higher score indicates a higher the level of depression. The overall scale has been shown to have excellent internal consistency (Cronbach’s α = 0.90) [34].

### Beck Anxiety Inventory (BAI)

The 21-item Chinese version of the Beck Anxiety Inventory (BAI) was used to assess caregivers’ anxiety [36]. The BAI is a brief somatic symptoms-focused screening tool that was specifically developed as a measure to discriminate between anxiety and depression [37]. Each BAI item is rated on a 4-point Likert scale ranging from 0 (not at all) to 3 (severe [“I could barely stand it”]) [38]. The total BAI score ranges from 0 to 63, where a higher score indicates a higher the level of anxiety. The overall scale has been shown to have excellent internal consistency (Cronbach’s α = 0.95, Guttman split-half coefficient = 0.91) [36].

### Neuropsychiatric Inventory (NPI)

The severity and frequency of behavioral and psychological symptoms of PWD were assessed using the Neuropsychiatric Inventory (NPI) [39]. The NPI comprises 12 domains that are symptoms associated with dementia (i.e., delusions, hallucinations, agitation, dysphoria, anxiety, apathy, irritability, euphoria, disinhibition, aberrant motor behavior, night-time behavior disturbances, and appetite and eating abnormalities). The NPI is a general outcome measure that is widely used to assess behavioral changes for patients with dementia [40]. The participants in the present study (i.e., caregivers of the PWD) were asked to rate the frequency of the symptoms of that domain on a scale ranging from 1 (occasionally, less than once per week) to 4 (very frequently, once, or more per day or continuously) and the severity of the same symptoms on a scale ranging from 1 (mild) to 3 (severe). The total NPI score ranges from 0 to 120, where a higher score indicates a higher the level of behavioral and psychological symptoms. The overall scale has been shown to have good internal consistency (Cronbach’s alpha 0.88 and a test–retest correlation of 0.79 for frequency and 0.86 for severity [41]).

## Data analysis

The data were analyzed using SPSS version 22 (SPSS, Chicago, Illinois, USA). Descriptive statistics were carried out to compute means and standard deviations as well as internal consistency. Bivariate Pearson’s correlations were used to assess the associations between study variables.

Ordinary least squares (OLS) regression analyses were performed, with neuropsychiatric symptoms as the predictor, affiliate stigma and caregiver burden as proposed mediators, and the severity of depression and anxiety as the outcome measures. Covariates in the regression models included age, sex, marital status, employment status, education, and relationship of caregiver, and PWD’s age, sex, marital status. The parallel mediation (Model 4 in Hayes’ PROCESS macro) and the sequential multiple mediation (Model 6 in Hayes’ PROCESS macro) of PROCESS macro developed by Hayes [42] were conducted. In the parallel mediation model, the mediating effect of PWDs’ NPI on caregiver depression or anxiety were examined through caregiver burden and affiliate stigma. Sequential multiple mediation analysis was used to determine the effects of mediators in the models. Bias-corrected bootstrap confidence interval (CI) based on 5000 bootstrapping samples with a 95% level of confidence was used to examine the mediation effects (i.e., indirect effects). When the confidence intervals do not include zero, the mediation effect is interpreted as significant.

## Results

Table 1 shows the socio-demographic and clinical characteristics of the 261 patient-caregiver dyads. Overall, the gender distribution was approximately equal for the caregivers with mean age of 52.9 years (SD ± 12.3). On average, the caregivers had received 11.2 years of education (SD ±4.2). Most of the caregivers were children of PWD (60.9%), had full-time employment (more than 30 hours per week) (52.5%), were married (78.2%), were living with PWD (70.9%), and were a primary caregiver (83.14%). Nearly half of caregivers (49.43%) self-reported caring for PWD for more than 8 hours a day on average, and 26.43% of them were caring for PWD all-day. Regarding the psychosocial characteristics of the caregivers, the mean score was 40.0 (out of 96) for caregiver burden (SD ± 19.1), 35.2 (out of 88) for affiliate stigma (SD ± 11.0), 12.7 (out of 54) for depression (SD ± 11.2), and 7.9 (out of 63) for anxiety (SD ± 8.8). Nearly two-thirds of the PWD (63.6%) were females with a mean age of 79.3 years (SD ± 6.8). Slightly more than half of the PWD were married (55.2%). Moreover, the mean score for neuropsychiatric symptoms of the PWD was 18.7 (out of 120) (SD ± 20.4); more than three-quarters of PWD (75.9%) had Clinical Dementia Rating Stage 0.5–1, with Stage 1 being the majority.

**Table 1:** Participant characteristics

	Mean ± SD	*n* (%)
**Caregivers**		
Age (in years)	52.92 ± 12.33	261 (100)
Years of education	11.22 ± 4.22	261 (100)
Sex		
Male		125 (47.9)
Female		136 (52.1)
Marital status		
Married		204 (78.2)
Single		36 (13.8)
Other		21 (8.1)
Employment status		
Full-time employment (>30 hours per week)		137 (52.5)
Part-time employment (≦30 hours per week)		6 (2.3)
Housekeeper		52 (19.9)
Retired		43 (16.5)
No employment		23 (8.8)
Relationship with PWD		
Children		159 (60.9)
Spouse		37 (14.2)
Other		65 (24.9)
Caregiver burden (range: 0–96)	39.95 ± 19.18	
Affiliate stigma (range: 22–88)	35.19 ± 10.99	
Depression (range: 0–54)	12.66 ± 11.22	
Anxiety (range: 0–63)	7.93 ± 8.80	
**People with dementia**		
Age (in years)	79.28 ± 6.78	
Sex		
Male		95 (36.4)
Female		166 (63.6)
Marital status		
Married		144 (55.2)
Widowed /Divorced /Single/Separated		117 (44.8)
Neuropsychiatry Inventory (range: 0–120)	18.74 ± 20.37	

Note, *PWD* People with dementia

Table 2 shows the bivariate correlations between the studied variables. The results showed that neuropsychiatric symptoms were positively associated with caregiver burden (r = .44, *p* < .01), affiliate stigma (r = .34, *p* < .01), caregiver depression (r = .36, *p* < .01) and with caregiver anxiety (r = .35, *p* < .01). Table 2 additionally demonstrates how neuropsychiatric symptoms, caregiver burden, affiliate stigma, depression, and anxiety associated with other demographic characteristic of caregivers and PWD.

**Table 2:** Pearson’s correlations among the study variables

Variables	1	2	3	4	5	6	7	8	9	10	11	12	13	14
1. NPI (X)	–													
2. CBI (M_1_)	0.44^**^	–												
3. ASS (M_2_)	0.34^**^	0.65^**^	–											
4. TDQ (Y_1_)	0.36^**^	0.67^**^	0.55^**^	–										
5. BAI (Y_2_)	0.35^**^	0.53^**^	0.49^**^	0.80^**^	–									
6. Cg Age	0.05	−0.15^*^	−0.03	0.00	0.01	–								
7. Cg Sex	0.16^**^	0.19^**^	0.02	0.21^**^	0.23^**^	−0.14^*^	–							
8. Cg Marital status	−0.04	0.06	−0.03	0.09	0.08	−0.30^**^	0.16^**^	–						
9. Cg Job	0.09	0.05	−0.03	0.09	0.14^*^	0.21^**^	0.26^**^	0.11	–					
10. Cg Years of education	−0.15^*^	0.09	0.01	−0.07	−0.03	− 0.55^**^	−0.16^**^	0.18^**^	−0.10	–				
11. Relation	− 0.08	0.08	−0.11	− 0.04	− 0.01	−0.70^**^	0.16^**^	0.15^*^	−0.05	0.46^**^	–			
12. PWD sex	−0.05	0.01	0.01	0.05	−0.00	−0.06	− 0.10	0.14^*^	0.04	0.10	0.11	–		
13. PWD Marital status	0.01	0.01	−0.01	− 0.02	−0.02	− 0.23^**^	0.02	0.23^**^	0.01	0.13*	0.33^**^	0.22^**^	–	
14. PWD age	−0.06	− 0.12	−0.26^**^	− 0.06	−0.02	0.16^**^	−0.02	0.01	0.09	0.08	0.27^**^	0.04	0.17^**^	–

Note. ^*^p < .05, ^**^p < .01, *NPI* Neuropsychiatry Inventory, *CBI* Caregiver Burden Inventory, *ASS* Affiliate stigma scale, *TDQ* Taiwanese Depressive Questionnaire, *BAI* Beck Anxiety Inventory, *Cg* caregiver, *PWD* People with dementia, *X* independent variable; M_1_ and M_2_: mediator; Y_1_ and Y_2:_ dependent variable

**Fig. 1:**
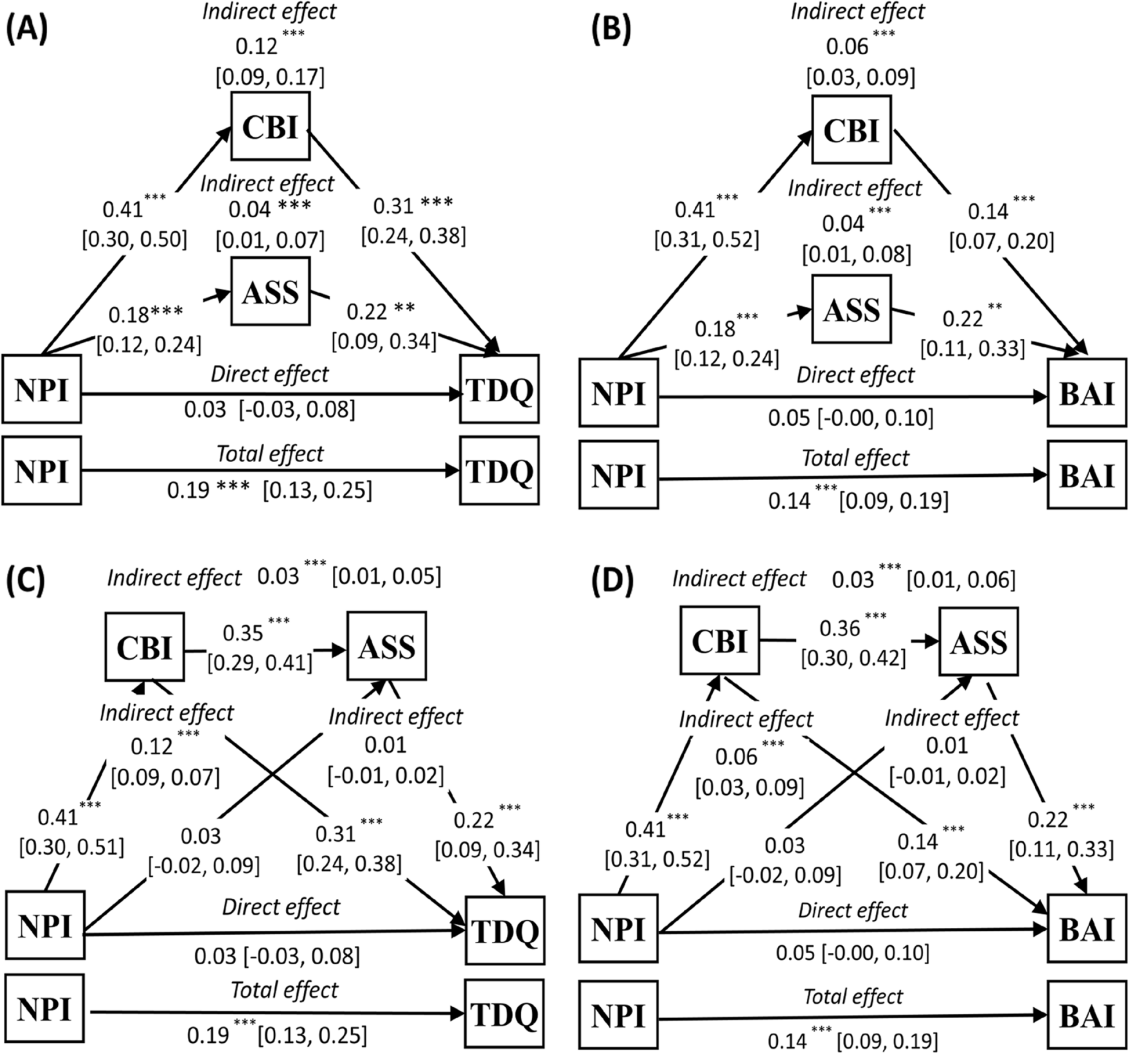
A Parallel mediation model with affiliate stigma (ASS) and caregiver burden (CBI) as mediators between behavioral and psychological symptoms (NPI) and depression (TPQ). **B** Parallel mediation model with ASS and CBI as mediators between NPI and anxiety (BAI). **C** Sequential mediation model with CBI and ASS as mediators between NPI and TPQ. **D** Sequential mediation model with CBI and ASS as mediators between NPI and BAI. All models controlled for age, sex, marital status, employment status, education and relationship of caregiver, and patient’s age, sex, marital status. * *p* <.05, ** *p* <.01, ***p<0.001

Four mediation models were carried out (Fig. 1 and Table 3). The first mediation model (Model A; Table 3 and Fig. 1 A) showed that the direct effect of PWDs’ neuropsychiatric symptoms on caregiver depression was not statistically significant (β = 0.03, *p* = .32) However, this mediation model showed a significant indirect path from PWDs’ neuropsychiatric symptoms to caregiver depression via caregiver burden (β = 0.12, 95% CI [0.09, 0.17]) and affiliate stigma (β = 0.04, 95% CI [0.01, 0.07]).

**Table 3:** Models of the effect of patient’s behavioral and psychological symptoms on mental health of caregivers with mediators of caregiver burden and affiliate stigma

Model				
(A)	Coefficient	SE	***t***	***p***
Total effect of NPI on TDQ (without accounting the potential mediators)	0.19	0.03	5.86	< 0.001
Direct effect of NPI on TDQ in mediated model	0.03	0.03	0.99	0.32
Indirect effect of NPI on TDQ	**Effect**	**Boot SE**	**Boot LLCI**	**Boot ULCI**
Total indirect effect	0.16	0.02	0.12	0.21
Indirect effect via CBI	0.12	0.02	0.09	0.17
Indirect effect via ASS	0.04	0.01	0.01	0.07
(B)	**Coefficient**	**SE**	***t***	***p***
Total effect of NPI on BAI (without accounting the potential mediators)	0.14	0.03	5.58	< 0.001
Direct effect of NPI on BAI in mediated model	0.05	0.03	1.95	0.05
Indirect effect of NPI on BAI	**Effect**	**Boot SE**	**Boot LLCI**	**Boot ULCI**
Total indirect effect	0.09	0.02	0.06	0.14
Indirect effect via CBI	0.06	0.01	0.03	0.09
Indirect effect via ASS	0.04	0.02	0.01	0.07
(C)	**Coefficient**	**SE**	***t***	***p***
Total effect of NPI on TDQ (without accounting the potential mediators)	0.19	0.03	5.63	< 0.001
Direct effect of NPI on TDQ in mediated model	0.03	0.03	0.99	0.32
Indirect effect of NPI on TDQ	**Effect**	**Boot SE**	**Boot LLCI**	**Boot ULCI**
Total indirect effect	0.16	0.02	0.12	0.21
Indirect effect via CBI	0.12	0.02	0.09	0.17
Indirect effect via ASS	0.01	0.01	−0.01	0.02
Indirect effect via CBI and ASS	0.03	0.01	0.01	0.05
(D)	**Coefficient**	**SE**	***t***	***p***
Total effect of NPI on BAI (without accounting the potential mediators)	0.14	0.03	5.73	< 0.001
Direct effect of NPI on BAI in mediated model	0.05	0.02	1.93	0.05
Indirect effect of NPI on BAI	**Effect**	**Boot SE**	**Boot LLCI**	**Boot ULCI**
Total indirect effect	0.10	0.02	0.07	0.14
Indirect effect via CBI	0.06	0.01	0.03	0.09
Indirect effect via ASS	0.01	0.01	−0.01	0.02
Indirect effect via CBI and ASS	0.03	0.01	0.01	0.06

*Boot* bootstrapping, *LLCI* lower limit confidence interval, *ULCI* upper limit confidence interval, *SE* standard error. (A) & (C) Unstandardized coefficients for the associations of affiliate stigma and caregiver burden with patient’s behavioral and psychological symptoms for model predicting depression of caregiver. (B) & (D) Unstandardized coefficients for the associations of affiliate stigma and caregiver burden with patient’s behavioral and psychological symptoms for model predicting anxiety of caregiver

The second mediation model (Model B; Table 3 and Fig. 1 B) showed that the direct effect of PWDs’ neuropsychiatric symptoms on caregiver anxiety was not statistically significant (β = 0.05, *p* = .054). However, this mediation model showed a significant indirect path from PWDs’ neuropsychiatric symptoms to caregiver anxiety via caregiver burden (β = 0.06, 95% CI [0.03, 0.09]) and affiliate stigma (β = 0.04, 95% CI [0.01, 0.07]).

The third mediation model (Model C; Table 3 and Fig. 1 C) showed that the direct effect of PWD’s neuropsychiatric symptoms on caregiver depression was not statistically significant (β = 0.03, *p* = .32). However, this sequential mediation model showed a significant indirect path from PWDs’ neuropsychiatric symptoms to caregiver depression via caregiver burden and affiliate stigma (β = 0.03, 95% CI [0.01, 0.05]). This model explained 52.34% of the variance in depression.

The final mediation model (Model D; Table 3 and Fig. 1 D) showed that the direct effect of PWD’s neuropsychiatric symptoms on caregiver anxiety was not statistically significant (β = 0.03, *p* = .05). However, this sequential mediation model showed a significant indirect path from PWDs’ neuropsychiatric symptoms to caregiver anxiety via caregiver burden and affiliate stigma (β = 0.06, 95% CI [0.03, 0.09]). This model explained 37.72% of the variance in anxiety.

## Discussion

The results of the present study supported the simplified Stress Process Model (SPM) that affiliate stigma and caregiver burden mediated the association of PWDs’ behavioral and psychological symptoms and caregivers’ mental health. More specifically, the present study examined two mediation models, and the significant association between NPI score and affiliate stigma (Fig. 1 A, Fig. 1 B) became nonsignificant when affiliate stigma was positioned after caregiver burden (Fig. 1 C, Fig. 1 D). To the best of the present authors’ knowledge, this is the first study to examine whether caregiver burden and affiliate stigma are mediators in the association between behavioral and psychological symptoms and caregiver mental health. These findings indicated that there could be a directional association between these two mediators, such that caregiver burden may reduce affiliate stigma, which subsequently may improve the psychological health of caregivers. However, the cross-sectional design in the present study cannot provide evidence for these proposed directions. Only longitudinal designs can corroborate the directions proposed in the present study.

Previous studies have shown that affiliate stigma has an important effect on caregiver burden and that the caregiver dimension of affiliate stigma has the greatest impact [43]. The results of the present study concur with the literature in the finding that caregiver burden is a significant predictor of affiliate stigma [11, 44]. In order to better understand the mediating role of affiliate stigma for caregivers of PWD, the present study analyzed both a parallel mediation model (Table 3 and Fig. 1 A, Fig. 1 B) and a sequential mediation model (Table 3 and Fig. 1 C, Fig. 1 D). Results from both models were generally comparable. However, the association between NPI score and affiliate stigma was not found in sequential mediation model, only the parallel mediation model. Parallel mediation analysis showed that the two mediators (caregiver burden, affiliate stigma) fully mediated the relationship between PWDs’ behavioral and psychological symptoms and caregiver mental health. However, while caregiver burden was found to significantly contribute to the overall indirect effect, affiliate stigma did not mediate the relationship between PWDs’ behavioral and psychological symptoms and caregiver mental health in the sequential mediation model.

The aforementioned findings indicate that there might be a directional association between these two mediators. More specifically, caregiver burden may reduce affiliate stigma, which may subsequently improve the psychological health of caregivers. Nevertheless, the effect of affiliate stigma on mental health for caregivers of PWD was consistent between the two models. Therefore, a tentative conclusion is that affiliate stigma is an important factor in caregiver burden, with caregiver burden as the primary mediator. The finding of affiliate stigma as a mediator suggests that providing support services to caregivers to improve their mental health could also reduce caregiver burden.

The results of the present study indicated that experiencing subjective caregiver burden was associated with increased risk of psychological distress. These findings are consistent with previous findings [45–47] and in line with SPM (i.e., primary stressors such as caregiver burden leads to mental health problems such as psychological distress) [12]. Moreover, caregivers of PWD are reported to have poorer mental health and a higher level of psychological distress than those who are caring for individuals with a physical disability [48, 49]. The health problems, especially mental health problems, among caregivers of PWD are likely to be explained by their caregiver burden. It is challenging for caregivers of PWD to take care of their family member’s disruptive behaviors comprising behavioral and psychological symptoms resulting from their dementia [50, 51]. Such caregiver burden may be additional to the feelings of embarrassment and shame, which are further associated with affiliate stigma.

The results of the present study concur with the prior findings [27, 28] that affiliate stigma is positively associated with depression and anxiety. Therefore, the positive relationships between affiliate stigma and psychological distress can be explained by the reason that caregivers of PWD feel themselves as inferior (i.e., endorse stigma in themselves) and the negative opinions toward themselves increase their mood problems, such as psychological distress. Accordingly, healthcare providers and social services may consider developing appropriate interventions to reduce affiliate stigma. Given that affiliate stigma was found to be associated with depression and anxiety, the reduction of affiliate stigma is needed to help caregivers improve their psychological distress. Subsequently, other benefits, such as improved outcomes for PWD, may also be gained.

### Limitations

The present study has some limitations. First, participants were from a convenience sample of PWD and their caregivers with referrals from the same area in Taiwan. Therefore, the findings may not be generalized to those who might not have been referred, because of the differences in types of dementia, need for caregiver assistance, and use of healthcare services. Second, the study was cross-sectional study and therefore causal relationships were unable to be determined. Future research should ideally comprise a longitudinal design, which can assess studied variables across time and provide stronger evidence for causal relationships. Third, the sample size in the present study was relatively small. However, the main analyses (i.e., the mediation models) were based on 5000 bootstrapped samples, which are robust and unlikely to have power issues [52]. However, given that the sample consisted of a small subset of kinship, future studies should separate these kinships of affiliate stigma in order to examine the distinct main effects.

## Conclusion

Despite these limitations, the present study provides new knowledge that caregiver burden and affiliate stigma were found to mediate the relationship between behavioral and psychological symptoms and caregivers’ psychological distress. Therefore, by considering mental health concerns in intervention or prevention measures in helping caregivers of PWD, it may provide more effective approaches to decrease psychological distress and potential negative consequences of the caregiver burden, especially in relation to their depression and anxiety. Future similar studies with bigger and more representative samples across different countries and cultures may help support the generalizability of the findings and better understand the mechanisms underlying the associations between dementia and affiliate stigma.

## Data Availability

The datasets generated and/or analyzed during the present study are not publicly available owing to patient privacy and ethical issues. However, they are available from the corresponding author upon reasonable request.

## Abbreviations

PWD: People with Dementia
CBI: Caregiver Burden Inventory
ASS: Affiliate Stigma Scale
TDQ: Taiwanese Depression Questionnaire
BAI: Beck Anxiety Inventory
AD: Alzheimer’s Disease
SPM: Stress Process Model
NPI: Neuropsychiatric Inventory
OLS: Ordinary Least Squares
CI: Confidence Interval
